# A comparison of zero-inflated and hurdle models for modeling zero-inflated count data

**DOI:** 10.1186/s40488-021-00121-4

**Published:** 2021-06-24

**Authors:** Cindy Xin Feng

**Affiliations:** Department of Community Health and Epidemiology, Faculty of Medicine, Dalhousie University, 5790 University Avenue, Halifax, B3H 4R2 Nova Scotia Canada

**Keywords:** Zero inflation, Hurdle model, Zero deflation, Model diagnosis

## Abstract

Counts data with excessive zeros are frequently encountered in practice. For example, the number of health services visits often includes many zeros representing the patients with no utilization during a follow-up time. A common feature of this type of data is that the count measure tends to have excessive zero beyond a common count distribution can accommodate, such as Poisson or negative binomial. Zero-inflated or hurdle models are often used to fit such data. Despite the increasing popularity of ZI and hurdle models, there is still a lack of investigation of the fundamental differences between these two types of models. In this article, we reviewed the zero-inflated and hurdle models and highlighted their differences in terms of their data generating processes. We also conducted simulation studies to evaluate the performances of both types of models. The final choice of regression model should be made after a careful assessment of goodness of fit and should be tailored to a particular data in question.

## Introduction

In public health and epidemiology research, count data with a large proportion of zeros are often encountered. For example, in health services utilization study, the number of service utilization often includes a large number of zeros representing the patients with no utilization during the study period. In the substance abuse field, substances of interest are characterized by different frequencies of drug or alcohol use with a large number of patients reporting zero days of use during treatment. A common feature of this type of data is that the count measure tends to have excessive zero beyond a common count distribution can accommodate, such as Poisson or negative binomial. For example, in counting the number of responses to a disease, an individual may have no disease response because of the individual is immune or resistant to the disease. Previous research has shown that if excessive zero is not accounted for, unreasonable fit for both the zeros and nonzero counts will be resulted (Perumean-Chaney et al. 2013). Zero-inflated (ZI) (Lambert 1992) and hurdle models(Mullahy 1986; Heilbron 1994) have been developed to model zero-inflation when the regular count models such as Poisson or negative binomial are unrealistic. Both types of models have gained increasing popularities in many fields including public health services research (Neelon et al. 2010; Neelon et al. 2013; Neelon et al. 2016), substance abuse (DeSantis and Bandyopadhyay 2011; Buu et al. 2012), occupational injury (Yau and Lee 2001), medicine (Bohning et al. 1999; Rose et al. 2006), psychology (Atkins and Gallop 2007), public health (Yau and Lee 2001; Yau et al. 2003; YB 2002; Sharker et al. 2020), ecological and environmental studies (Agarwal et al. 2002; Rathbun and Fei 2006; Feng and Dean 2012; Feng 2020).

Despite the increasing popularity of ZI and hurdle models, the differences between these two types of models are understudied. The choice between the two types of models is often determined by comparing model fit statistics post-fitting both types of models. Among these studies, the conclusions are inconsistent. Some revealed that ZI and hurdle models are indistinguishable with respect to goodness of fit measures (Xu et al. 2015; Tüzen et al. 2018); whereas, some studies found the hurdle model had a better fit than the ZI model (Min and Agresti 2005; Sharker et al. 2020) and other empirical application found ZI model performs better than the hurdle model (Hu et al. 2011). It is therefore desired to identify the situations where hurdle models perform better than ZI and vice versa through simulation studies. Model comparison measures, such as Akaike information criterion (AIC) (Akaike et al. 1973; Akaike 2011) and Vuong’s test (Vuong 1989) are used to compare the goodness of fit of these two types of models. Examination of residuals has been an important step to detect model misspecification and departure from the model assumption. Randomized quantile residuals (RQR) have been proposed by Dunn and Smyth (Dunn and Smyth 1996) for assessing the model fits for discrete outcome data. If the model is correctly specified, RQRs should be approximately normally distributed and the plot of RQRs against the predicted values should be randomly scattered without any discernible pattern(Dunn and Smyth 1996; Feng et al. 2020). Built on these works, we examine and compare the absolute fit (how well the model fits the data) of the ZI and hurdle models using RQRs.

The paper is organized as follows. In Section 2, we give a brief review of hurdle and ZI regression models. Section 3 reviewed model comparison strategies for examining model adequacy. Section 4 presented simulation studies to compare hurdle and ZI models. Concluding remarks are given in Section 5.

## Statistical models

### 2.1 Zero-Inflated model

In a zero-inflated (ZI) model (Lambert 1992), zero observations have two different origins: “structural” and “sampling”. The sampling zeros are from the usual Poisson or negative binomial (NB) distribution, which are assumed that were occurred by chance.

The general structure of a ZI model is given as:

1 $$\begin{array}{@{}rcl@{}} P(Y_{i}=y_{i})= \left\{ \begin{array}{ll} \pi_{i}+(1-\pi_{i})p(y_{i}=0; \mu_{i}) & y_{i}=0,\\ (1-\pi_{i})p(y_{i}; \mu_{i}) & y_{i}>0, \end{array} \right.  \end{array} $$

which consists of a degenerate distribution at zero and an untruncated count distribution with a vector of parameters *μ*_*i*_. If the count distribution follows a Poisson distribution, the *zero inflated Poisson model (ZIP)* is given by:

2 $$\begin{array}{@{}rcl@{}} P(Y_{i}=y_{i})&=&\left\{ \begin{array}{ll} \pi_{i}+\left(1-\pi_{i}\right)e^{-\mu_{i}} & \text{if \(y_{i}=0\)},\\ (1-\pi_{i})\frac{e^{-\mu_{i}}\mu_{i}^{y_{i}}}{y_{i}!} & \text{if \(y_{i}>0\)} \end{array} \right.,  \end{array} $$

where *μ*_*i*_ is the mean of the standard Poisson distribution. ZI model is also fumulated as a latent variable model with an unobserved Bernouli random variable *z*_*i*_ (Lambert 1992):

3 $$\begin{array}{@{}rcl@{}} z_{i}= \left\{ \begin{array}{ll} 1 & \text{if} y_{i} \text{is structural zero},\\ 0 & \text{if} y_{i} \sim \text{Poisson}(\mu_{i}), \end{array} \right.  \end{array} $$

which establishes

4 $$\begin{array}{@{}rcl@{}} E(y_{i})&=&E\left(E(y_{i}|z_{i})\right)=(1-\pi_{i})\mu_{i} \end{array} $$

5 $$\begin{array}{@{}rcl@{}} Var(y_{i})&=&E(Var(y_{i}|z_{i}))+Var(E(y_{i}|z_{i}))=(1-\pi_{i})\mu_{i}\left(1+\mu_{i}\pi_{i}\right). \end{array} $$

For modeling the count component of a ZI model, Poisson regression assumes the conditional mean equals to the conditional variance, which may not be valid in some situations. If data have greater conditional variance than is assumed under the Poisson model, overdispersion would occur, which may be due to population heterogeneity or clustering, omission of important covariates in the model, or the presence of outliers (Cox 1983; Dean 1992; Dean and Lundy 2016; Payne et al. 2017). Negative binomial (NB) regression could be then used to model overdispersed Poisson count data. The *zero inflated negative binomial model (ZINB)* is then given by:

6 $$\begin{array}{@{}rcl@{}} P(Y_{i}=y_{i})&=&\left\{ \begin{array}{ll} \pi_{i}+(1-\pi_{i})\left[\left(\frac{r}{\mu_{i}+r}\right)^{r}\right] & \text{if \(y_{i}=0\)},\\ (1-\pi_{i})\frac{\Gamma(y_{i}+r)}{\Gamma(r)y_{i}!} \left(\frac{\mu_{i}}{\mu_{i}+r}\right)^{y_{i}} \left(\frac{r}{\mu_{i}+r}\right)^{r} & \text{if \(y_{i}>0\)} \end{array} \right.,  \end{array} $$

where *μ*_*i*_ is the mean of the NB model, *π*_*i*_ is the probability of a structural zero, *r* is the dispersion parameter, *Γ* is the gamma function. The mean and variance of the ZINB are then given by *E*(*y*_*i*_)=(1−*π*_*i*_)*μ*_*i*_ and *Var*(*y*_*i*_)=(1−*π*_*i*_)*μ*_*i*_(1+*μ*_*i*_/*r*+*π*_*i*_*μ*_*i*_). As *r* goes to infinity, the ZINB reduces to the ZIP model. Therefore, small values of *r* indicate overdispersion. In many applications, it is common to assume that the parameters *μ*_*i*_ and *π*_*i*_ depend on vectors of explanatory variables ***x***_*i*_ and ***z***_*i*_. Covariates can be associated with the probability of a structural zero, *π*_*i*_, as well as the mean function *μ*_*i*_ of the count model. Generally, *π*_*i*_ is modeled with a logistic regression and *μ*_*i*_ is modeled as a log-linear regression. The ZI model can be written as,

7 $$\begin{array}{@{}rcl@{}} \log (\mu_{i})=\boldsymbol{x}_{i}^{T}\boldsymbol{\alpha}, \text{logit}(\pi_{i}) =\boldsymbol{z}_{i}^{T}\boldsymbol{\beta} \end{array} $$

where ***α*** and ***β*** are regression coefficients for the covariates $\boldsymbol {x}_{i}^{T}$ and $\boldsymbol {z}_{i}^{T}$. Note that the explanatory variables describing the *μ*_*i*_ do not need to be the same as those describing *π*_*i*_. The ZINB model allows for added flexibility compared to the ZIP model. It allows for over-dispersion arising from excess zeros and heterogeneity in the Poisson component, whereas the ZIP model only accommodates over-dispersion from excess zeroes.

### 2.2 Hurdle model

In contrast to ZI models, hurdle models (Mullahy 1986; Heilbron 1994) can be viewed as a two-component mixture model consisting of a zero mass and the positive observations component following a truncated count distribution, such as truncated Poisson or truncated NB distribution.

Let *Y*_*i*_ denote the response of the *i*th observation, *i*=1,⋯,*n*, where *n* denote the total number of observations. The general structure of a hurdle model is given by

8 $$\begin{array}{@{}rcl@{}} P(Y_{i}=y_{i})= \left\{ \begin{array}{ll} p_{i} & y_{i}=0,\\ (1-p_{i})\frac{p(y_{i}; \mu_{i})}{1-p(y_{i}=0; \mu_{i})} & y_{i}>0, \end{array} \right.  \end{array} $$

where *p*_*i*_ is the probability of a subject belonging to the zero component; *p*(*y*_*i*_;*μ*_*i*_) represents a probability mass function (PMF) for a regular count distribution with a vector of parameters *μ*_*i*_ and *p*(*y*_*i*_=0;*μ*_*i*_) is the distribution evaluated at zero. It can be seen that the positive count is governed by a regular counts distribution as the PMF divided by 1 minus the PMF of this regular counts distribution evaluated at zero.

For example, if the count distribution follows a Poisson distribution, the probability distribution for the *hurdle Poisson model* is written as:

9 $$\begin{array}{@{}rcl@{}} P(Y_{i}=y_{i})&=&\left\{ \begin{array}{ll} p_{i} & {y_{i}=0},\\ (1-p_{i})\frac{e^{-\mu_{i}}\mu_{i}^{y_{i}}/y_{i}!}{1-e^{-\mu_{i}}} & {y_{i}>0} \end{array} \right..  \end{array} $$

Alternatively, the non-zero count component can follow other distributions to account for overdispersion and NB distribution is the most commonly used. The *HNB* model is then given by:

10 $$\begin{array}{@{}rcl@{}} P(Y_{i}=y_{i})&=&\left\{ \begin{array}{ll} p_{i} & {y_{i}=0},\\ \frac{1-p_{i}}{1-\left(\frac{r}{\mu_{i}+r}\right)^{r}}\frac{\Gamma(y_{i}+r)}{\Gamma(r)y_{i}!} \left(\frac{\mu_{i}}{\mu_{i}+r}\right)^{y_{i}} \left(\frac{r}{\mu_{i}+r}\right)^{r} & {y_{i}>0} \end{array} \right.,  \end{array} $$

Similar as a ZI model, covariates can enter the probability of a zero *p*_*i*_ and the mean function *μ*_*i*_ for a hurdle model. Hence, the hurdle model can be written as:

11 $$\begin{array}{@{}rcl@{}} \log (\mu_{i})=\boldsymbol{x}_{i}^{T}\boldsymbol{\alpha}, \text{logit}(p_{i}) =\boldsymbol{z}_{i}^{T}\boldsymbol{\beta} \end{array} $$

where ***α*** and ***β*** are the regression coefficients for the covariates ***x***_*i*_ and ***z***_*i*_, respectively.

### 2.3 Hurdle model versus zero-Inflated model

In general, ZI and hurdle models differ based on their conceptualization of the zeros and interpretation of model parameters. A ZI model (Lambert 1992) assumes that zero counts result from a mixture of two distributions, one where subjects always produce zero counts, which are often called “structural zeros” or “excessive zeros”. Subjects who are exposed to the outcome but did not or did not report the experience of the outcome during the study period, are termed as “sampling zeros”. The rationale for differentiating the zeros into two groups is that excessive zeros are often due to the existence of a subpopulation of subjects who are not at risk for certain outcomes during the study period. For example, when modeling the count of certain high-risk behaviors, some participants may score zero because they are not at risk for such health-risk behavior; these are the structural zeros since they cannot exhibit such high-risk behaviors. Other participants who are at risk may score zero because they did not exhibit such high-risk behaviors during the study period. The likelihood of being from either population is estimated with a zero-inflation probability component, while the counts in the second population of the user group are modeled by an ordinary count distribution, such as a Poisson or negative binomial (NB) distribution. In contrast, a hurdle model (Mullahy 1986; Heilbron 1994) assumes all zero data are from one “structural” source with one part of the model being a binary model for modeling whether the response variable is zero or positive, and another part using a truncated model, such as a truncated Poisson or a truncated NB distribution for the positive data. For example, in healthcare utilization studies, the zero part involves the decision of seeking care, and the positive component determines how frequent the utilization among the user’s group. Below details of the difference between hurdle and zero-inflated models in terms of their ability to handle zero deflation and differences in the generating process for excessive zeros versus sampling zeros.

#### 2.3.1 Ability to handle zero deflation

Another important difference between hurdle and ZI models is their capacity to handle zero deflation (fewer zeros than expected by the data-generating process). ZI models are not able to handle zero-deflation at any level of a factor and will result in parameter estimates of infinity for the logistic component, whereas hurdle models can handle zero-deflation (Min and Agresti 2005).

As shown in Eq. (3), ZI models are only suitable for handling zero inflation, since the probability of observing zeros in a ZI model is always greater than the probability of sampling zeros, i.e.,

12 $$\begin{array}{@{}rcl@{}} \pi_{i}+(1-\pi_{i})p(y_{i}=0; \mu_{i})> p(y_{i}=0; \mu_{i}). \end{array} $$

In contrast, hurdle model is not only able to handle zero inflation, but also suitable for modelling zero deflation. As shown in Eq. (8), when the probability of observing any zeros is greater than the probability of observing sampling zeros, i.e., *p*_*i*_>*p*(*y*_*i*_=0;*μ*_*i*_) the data are zero inflated; whereas, when *p*_*i*_<*p*(*y*_*i*_=0;*μ*_*i*_), the data are zero-deflated. For example, when the true model is a HNB model, zero deflation occurs when

13 $$\begin{array}{@{}rcl@{}} \frac{\text{exp}\left(\boldsymbol{z}_{i}^{T}\boldsymbol{\beta}\right) }{1+{\text{exp}\left(\boldsymbol{z}_{i}^{T}\boldsymbol{\beta}\right) }}<\left(\frac{r}{\text{exp}\left(\boldsymbol{x}_{i}^{T}\boldsymbol{\alpha} \right)+r}\right)^{r}, \end{array} $$

which indicates that in zero-inflated count data, zero deflation could still occur at specific levels of covariates. Therefore, it is plausible that the hurdle model outperforms the counterpart ZI model, as the percentage of zero deflation across all the data points increases. As shown in Eq. (13), percentage of zero deflation depends on the mean structures for both the logistic and log-linear components. For illustration, we simulate data from a HNB model as follows,

14 $$\begin{array}{@{}rcl@{}} \text{logit}(p_{i}) =\beta_{0}+\beta_{1}x_{i}, \log (\mu_{i})=\alpha_{0}+\alpha_{1}x_{i} \end{array} $$

where *x*_*i*_ is a Bernoulli random variable with probability parameter 0.5. We also consider another scenario when *x*_*i*_ is generated from a standard normal distribution *N*(0,1). We set sample size as *n*=300, the intercept for both the zero and truncated counts components as *β*_0_=*α*_0_=1 to ensure the data are overall zero inflated. The regression coefficients of *x*_*i*_ for the zero (*β*_1_) and positive counts components (*α*_1_) are set as -2 to 2 at an increment of 0.02. Percentage of zero deflation across all the data points is then calculated as:

15 $$\begin{array}{@{}rcl@{}} \sum_{i=1}^{n} I \left\{\frac{\text{exp}(\beta_{0}+\beta_{1}x_{i}) }{1+{\text{exp}(\beta_{0}+\beta_{1}x_{i}) }}<\left(\frac{r}{\text{exp}(\alpha_{0}+\alpha_{1}x_{i})+r}\right)^{r} \right\}/n \end{array} $$

where *I*{·} is an indicator variable.

**Fig. 1:**
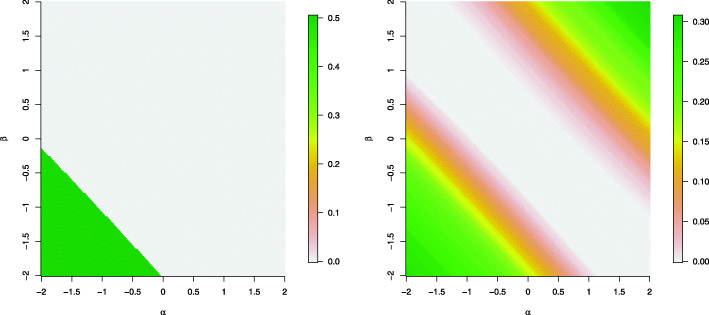
Percentage of zero deflation over all data points when the data are simulated from a HNB model of sample size *n*=300. In the left panel, the covariate *x* is a binary variable simulated from a Bernoulli distribution with probability parameter 0.5. In the right panel, the covariate is a continuou variable simulated from a standard normal distribution

The left panel of Fig. 1 displays the percentage of zero deflation as a function of the regression coefficients (*β* and *α*) in the two model components when the data are simulated from a HNB model with a binary covariate simulated from a Bernoulli distribution with probability parameter 0.5. As displayed, when the regression coefficients for the logistic component (*β*) and log-linear components (*α*) are below zero, more than 50% of the data are zero-deflated, shown as the green shaded areas in the bottom left corner.

**Fig. 2:**
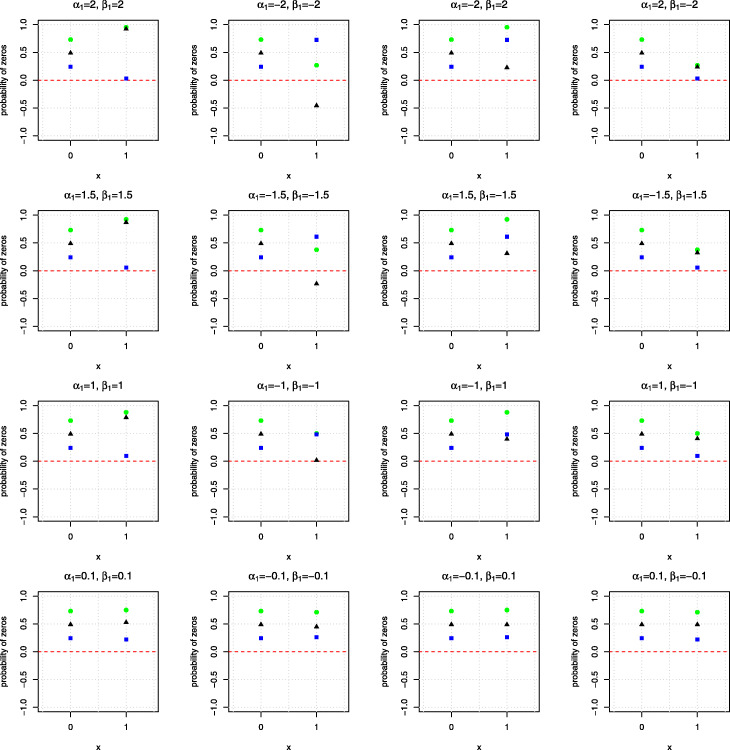
Probabilities of observing a zero (green), a sampling zero (blue) and their differences (black) against the covariate when the data are simulated from a HNB model with a binary covariate of sample size *n*=300. The intercepts for both the zero and truncated counts components are set as 1. The regression coefficients of the covariate for the zero (*β*_1_) and the truncated counts component (*α*_1_) are set as -2, -1.5, -1, -0.1, 0.1, 1, 1.5 and 2

To further demonstrate at what level of the covariate, zero deflation may occur, Fig. 2 plots the probability of being a zero, the probability of being a sampling zero, and their differences, i.e., probability of being a zero minus the probability of being a sampling zero against the covariate when the regression coefficients for the zero (*β*_1_) and the truncated counts component (*α*_1_) are set as -2, -1.5, -1, -0.1, 0.1, 1, 1.5 and 2. As shown in Fig. 2, when the covariate equals zero, no zero deflation would occur. Specifically, the probability of being a zero is exp(1)/(1+exp(1))≈0.75 and the probability of being a sampling zero from the NB model is (*r*/exp(1)+*r*)^*r*^≈0.24, when *r*=1.2. Therefore, when the covariate is zero, the probability of being zero is always greater than the probability of being a sampling zero in this setting. Nevertheless, when the binary covariate equals one, zero deflation occurs when *α*_1_ and *β*_1_ approach to -2, since the probability of being zero is exp(−1)/(1+exp(−1))≈0.27 and the probability of being a sampling zero is (*r*/exp(1)+*r*)^*r*^≈0.73; that is, the probability of being zero is less than the probability of being a sampling zero. In this illustrative example, the covariate was generated with 50% of ones, so the probability of zero deflation would be approximately equal to 50% when the regression coefficients *α* and *β* belong to the bottom left corner of Fig. 1. Similarly, if *q%* of one’s in the covariate, we would expect *q%* of zero deflation when *α*_1_ and *β*_1_ belong to the bottom left corner of Fig. 1.

The right panel of Fig. 1 displays the percentage of zero deflation as a function of the regression coefficients in the two model components in the scenario when the data are simulated from a HNB model with a continuous covariate generated from a standard normal distribution. As displayed, as the regression coefficients become larger in magnitude in the same direction, the percentage of zero deflation increases. For example, when *β*=−2 and *α*=−2 or when *β*=2 and *α*=2, the percentage of zero-deflation is above 30%. However, when the regression coefficients are in different sign, for example, *β*=2 and *α*=−2 or *β*=−2 and *α*=2, the percentage of zero deflation tends to be low.

**Fig. 3:**
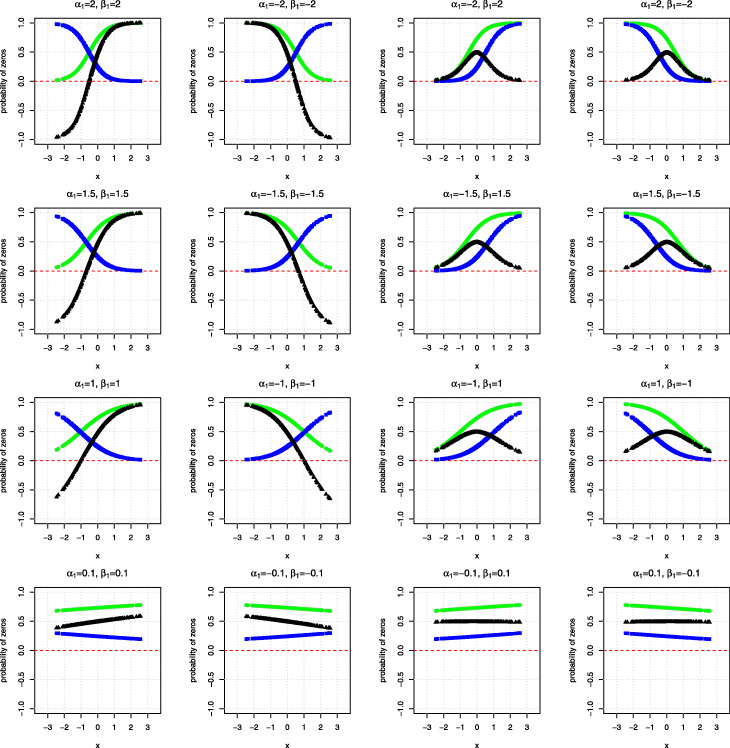
Probabilities of observing a zero (green), a sampling zero (blue) and their differences (black) against the covariate, when the data are simulated from a HNB model with a continuous covariate of sample size *n*=300. The intercepts for both the zero and truncated counts components are set as 1. The regression coefficients of the covariate for the zero (*β*_1_) and the truncated counts component (*α*_1_) are set as -2, -1.5, -1, -0.1, 0.1, 1, 1.5 and 2

To further explore at what level of covariate zero deflation may occur, Fig. 3 plots the probability being a zero, the probability being sampling zeros, and their differences against the covariate when the regression coefficients for the logistic component (*β*_1_) and the log-linear component (*α*_1_) are set as -2, -1.5, -1, -0.1, 0.1, 1, 1.5 and 2. As shown in the Figure, when the regression coefficients for the logistic and log-linear components are equal to 2, zero deflation occurs when the covariate *x* is roughly below -0.5. In this case, the means of the logistic and log-linear components are negative, resulting in a small chance of observing zeros but a large chance of observing sampling zeros. Similarly, when *α*_1_ and *β*_1_ are equal to −2, zero deflation is observed when the covariate *x* is above 0.5. However, when the signs of *α*_1_ and *β*_1_ are opposite, or both are at a relatively smaller magnitude, zero deflation will be less likely to occur.

In the circumstances when there is no zero deflation at any level of the covariates, ZI model can be rewritten as a hurdle model. To illustrate this, suppose a simple hurdle model is written as follows,

16 $$\begin{array}{@{}rcl@{}} \text{logit}(p_{i}) =\beta_{0}+\beta_{1}x_{i} \log (\mu_{i})=\alpha_{0}+\alpha_{1}x_{i} \end{array} $$

where *x*_*i*_ follows a standard normal distribution *N*(0,1). The counterpart ZI model is expressed as

17 $$\begin{array}{@{}rcl@{}} \text{logit}(\pi_{i}) =\beta^{\ast}_{0}+\beta^{\ast}_{1}x_{i} \log (\mu_{i})=\alpha^{\ast}_{0}+\alpha^{\ast}_{1}x_{i} \end{array} $$

The connection between ZI and hurdle models can be built through equating the probability of observing zeros in the data, i.e.,

18 $$\begin{array}{@{}rcl@{}} \pi_{i}+(1-\pi_{i})p(0; \mu_{i})=p_{i} \end{array} $$

19 $$\begin{array}{@{}rcl@{}} \pi_{i}=\frac{p_{i}-p(0; \mu_{i})}{1-p(0; \mu_{i})}. \end{array} $$

When $\phantom {\dot {i}\!}x_{i}=0, p_{i}=e^{\beta _{0}}/\left (1+e^{\beta _{0}}\right)$, so

20 $$\begin{array}{@{}rcl@{}} \beta^{\ast}_{0}=\text{logit}(\pi_{i})=\text{logit}\left(\frac{e^{\beta_{0}}/\left(1+e^{\beta_{0}}\right)-p(0; \mu_{i})}{1-p(0; \mu_{i})}\right). \end{array} $$

When $\phantom {\dot {i}\!}x_{i}=1, p_{i}=e^{\beta _{0}+\beta _{1}}/\left (1+e^{\beta _{0}+\beta _{1}}\right)$, so

21 $$\begin{array}{@{}rcl@{}} \beta^{\ast}_{1}=\text{logit}(\pi_{i})-\beta^{\ast}_{0}=\text{logit}\left(\frac{e^{\beta_{0}+\beta_{1}}/\left(1+e^{\beta_{0}+\beta_{1}}\right)-p(0; \mu_{i})}{1-p(0; \mu_{i})}\right)-\beta^{\ast}_{0}. \end{array} $$

For example, for a HNB model of sample size *n*=1000. Suppose *β*_0_=*β*_1_=*α*_0_=*α*_1_=1. The intercept and regression coefficient for the ZINB model are then about $\beta ^{\ast }_{0}=0.599$ and $\beta ^{\ast }_{1}=1.288$. The second part of the hurdle model has the same parameters as the second part of the ZI model.

#### 2.3.2 Generating processes for excessive zeros versus sampling zeros

Although the hurdle model is able to handle zero deflation at any level of the covariates, it treats all the zeros generated from the same processes; whereas, the ZI model allows for two data generating processes for zeros depending on the mean structures of the logistic and log-linear components. As a result, it should be expected that the ZI model outperforms the hurdle model when the data generating processes for the excessive zeros and sampling zeros differ to some extent.

To measure the difference in probability of a binary variable being an excessive zero versus being a sampling zero, we employed the standardized difference for a binary random variable (Austin 2009), which is defined as

22 $$\begin{array}{@{}rcl@{}} d_{i}=\frac{\pi_{i1}-\pi_{i2}}{\sqrt{\frac{(\pi_{i1}(1-\pi_{i1})+\pi_{i2}(1-\pi_{i2})}{2}}}, \end{array} $$

where *π*_*i*1_ and *π*_*i*2_ denote the probability of the underlying Bernoulli distribution of the binary variable, i.e., the probability of being an excessive zero and sample zero, respectively. It should be noted that there is no accepted threshold for the standardized difference to indicate the presence of meaningful imbalance (Austin 2009). Nevertheless, a *α%* confidence interval for *d*_*i*_: *d*_*i*_±*z*_*α*/2_×*σ*(*d*_*i*_), where $\sigma (d_{i})=\sqrt {2+d_{i}^{2}/4}$ for comparing the means of Bernoulli variables (Hedges and Olkin 1985) was applied to approximately determine the differences in the generating process of sampling zeros and structural zeros. In this circumstance, the direction of the comparison is not of interest, but rather the magnitude of the differences, i.e., |*d*_*i*_|. As a result, at *α%* level of significance, if the absolute value of the standardized difference |*d*_*i*_| exceeds *z*_*α%*_×*σ*(*d*_*i*_), we regard there is strong evidence of the probabilities of being an excessive zero and sampling zero are substantially different. To quantify the overall distances between the probability being an excessive zero vs. sampling zero across all data points, we calculate the mean of |*d*_*i*_|>*z*_*α%*_×*σ*(*d*_*i*_).

To illustrate the impact of this standardized distance measure on the model fit performance between ZINB and hurdle models, we simulate data from a ZINB model with the mean structures as follows:

23 $$\begin{array}{@{}rcl@{}} \text{logit}(\pi_{i1}) =\beta_{0}+\beta_{1}x_{i}, \log (\mu_{i})=\alpha_{0}+\alpha_{1}x_{i} \end{array} $$

where *x*_*i*_ is a Bernoulli random variable with probability of event as 0.5. In another simulation scenario, *x* is generated from a standard normal distribution *N*(0,1). We set sample size as *n*=300, the intercept for both the zero and truncated counts components as *β*_0_=*α*_0_=1 and the regression coefficients of *x*_*i*_ for the zero (*β*_1_) and positive counts components (*α*_1_) as -2 to 2 at an increment of 0.02. In this illustrative example, $\pi _{i1}=\frac {\text {exp}(\beta _{0}+\beta _{1}x_{i}) }{1+{\text {exp}\left (\beta _{0}+\beta _{1}x_{i}\right) }}$ and $\pi _{i2}=p(y=0; \mu _{i})=\left (\frac {r}{\text {exp}(\alpha _{0}+\alpha _{1}x_{i})+r}\right)^{r}$.

We then calculate the mean of *d*_*i*_,*i*=1⋯,*n* as the measure of discrepancy of the data generating processes between the excessive zero and sampling zero.

**Fig. 4:**
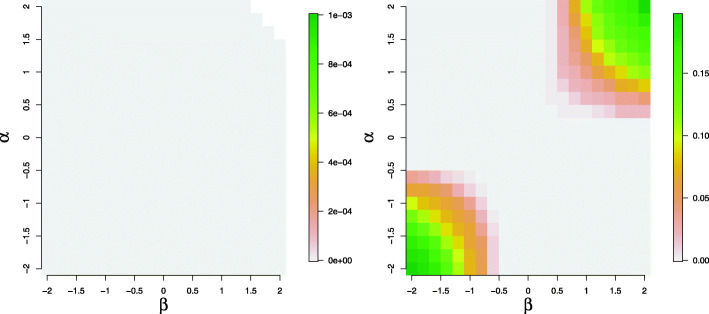
Mean of the standardized differences of the probability of being an excessive zero and the probability of being a sampling zero when data are simulated from a ZINB model of sample size *n*=300. In the left panel, the covariate is a binary variable from a Bernoulli random variable with probability parameter 0.5. In the right panel, the covariate is a continuous variable from a standard normal distribution

Figure 4 displays the mean of *d* over varying values of the regression coefficients of the two model components when the data are simulated from a ZINB model with a binary covariate generated from a Bernoulli random variable with probability parameter 0.5 (left panel) and a ZINB model with a continuous covariate generated from a standard normal distribution (right panel). As shown in the left panel of Fig. 4, the difference in the probabilities of being an excessive zero versus being a sampling zero is negligible at various levels of the covariate effects; whereas, under the scenario with a continuous standard normal covariate (right panel of Fig. 4), the difference in the probabilities of observing an excessive zero versus observing a sampling zero manifests when the regression coefficients of the covariate in the logistic and log-linear components approach -2 or 2.

## Model selection and goodness-of-fit statistic

### 3.1 Relative fit measures

Akaike information criterion (AIC) (Akaike et al. 1973; Akaike 2011) is used for comparing the model fits between hurdle and ZI models, which is computed as *AIC*=−2log(*L*)+2*q*, where *L* is the likelihood, and *q* is the number of parameters in the model. In general, the best fitting model yields the lowest AIC values. Another widely used tool for comparing ZI versus hurdle models is Vuong test (Vuong 1989), which compares the likelihood functions between two models. Let *f*_1_(*y*_*i*_|*θ*_1_) and *f*_2_(*y*_*i*_|*θ*_2_) denote the probability distribution functions of two models. Their likelihood functions should be nearly identical when the two models fit the data equally well. Differences between the likelihood functions indicate which model fits the data better. Let $\hat {\theta }_{1} $ and $\hat {\theta }_{2}$ be the maximum likelihood estimate (MLE) of *θ*_1_ and *θ*_2_. The Vuong test is to compare the likelihood function at the MLE between the two models, that is $\rho _{i}=\text {log}(f_{1}(y_{i}|\hat {\theta }_{1}))-\text {log}(f_{2}(y_{i}|\hat {\theta }_{2}))$. The Vuong test for comparing $f_{1}(y_{i}|\hat {\theta }_{1})$ and $f_{2}(y_{i}|\hat {\theta }_{2})$ is then defined as $V=\sqrt {n}\bar {\rho }/s_{\rho }$, where $\bar {\rho }$ and *s*_*ρ*_ is the mean and standard deviation of the vector of ***ρ***=(*ρ*_1_,⋯,*ρ*_*n*_). Under the null hypothesis, i.e., the two models fit the data equivalently, Vuong’s statistic asymptotically follows a standard normal distribution. At the 5% level of significance, the critical value is 1.96, so if *V*>1.96, the statistic favours the model in the numerator; whereas, if *V*<−1.96, the statistic favours the model in the denominator, and when *V*∈(−1.96,1.96), two models fit the data equally, with no preference given to either model.

### 3.2 Absolute fit measures

Model checking is an essential step of statistical modeling that ensures the assumptions are met for valid inference. Residual diagnosis is often used to assess how well the model captures the characteristics of the data; however, when data is discrete and skewed, standard residuals such as Pearson and deviance residuals do not follow a standard normal distribution. A tool that quantifies model fit on an easy-to-understand aligning with the scale of the traditional residuals used in normal regression, would be helpful to practitioners.

Randomized quantile residual (RQR) was defined by Dunn and Smyth (1996) to diagnose counts models, such as Poisson or NB models, but has not been often used for diagnosing ZI or hurdle models. RQR has recently been demonstrated and evaluated through comprehensive simulation studies. The results showed that RQRs could be applied to diagnose regression models for scalar *y*_*i*_ provided that one can compute the CDF and PMF of the considered model (Feng et al. 2020). RQR can be defined as follows in general. Suppose we consider fitting a regression model with *F*(*y*_*i*_;*μ*_*i*_,*ϕ*) denoting the CDF for a response variable *y*_*i*_ given a set of covariates *x*_*i*_, where *μ*_*i*_ is typically a function of *x*_*i*_, for example the conditional mean of *y*_*i*_, whereas *ϕ* does not depend on *x*_*i*_, for example dispersion parameter. Let *d*(*y*_*i*_;*μ*_*i*_,*ϕ*) be the corresponding PMF of *F*(*y*_*i*_;*μ*_*i*_,*ϕ*). If *F* is discrete, the estimated lower tail probability is randomized into a uniform random number, which is defined as a function with a random number *u*_*i*_ from the uniform distribution on (0,1] as an additional argument, $ F^{\ast } (y_{i};\hat \mu _{i},\hat \phi, u_{i}) = F(y_{i}-; \hat \mu _{i},\hat \phi)+u_{i} d(y_{i};\hat \mu _{i},\hat \phi), $ where $F(y_{i}-;\hat \mu _{i},\hat \phi)$ is the lower limit of *F* at *y*_*i*_, i.e., $\sup _{y < y_{i}} F(y;\hat \mu _{i},\hat \phi)$, the lower limit in the “gap" of $F(\cdot, \hat \mu _{i},\hat \phi)$ at *y*_*i*_. Here we use right close interval for *u*_*i*_ only for mathematical convenient in our proof, which does not have practical implication. An alternative way to define the randomized lower tail probability as a uniform random number between $a=\sup _{y < y_{i}} F(y;\hat \mu _{i}, \hat \phi)$ and $b = F(y_{i}; \hat \mu _{i}, \hat \phi)$ (Dunn and Smyth 1996).

RQR for *y*_*i*_ is the standard normal quantile corresponding to the random lower tail probability with *μ*_*i*_ and *ϕ* estimated from the sample, $ q_{i} = \Phi ^{-1}(F^{\ast } (y_{i};\hat {\mu }_{i},\hat {\phi }, u_{i}))= q(y_{i};\hat {\mu }_{i},\hat {\phi _{i}},u_{i}), $ where *Φ*^−1^ is the quantile function of a standard normal distribution, and *u*_*i*_ is a random number uniformly distributed on (0,1]. *F*^∗^(*y*_*i*_;*μ*_*i*_,*ϕ*,*u*_*i*_) can be converted to any other standard distribution as above. The normal distribution is chosen because most people are familiar with normal random variates with the so-called “empirical rules”. For evaluating model goodness of fit, we can test the following hypotheses *H*_0_: Model fits the data well and *H*_*a*_: Model does not fit the data well, by examining the normality of RQR based on the Shapiro-Wilk (SW) normality test. Under the true model, the null hypothesis should not be rejected, so RQR should be normally distributed, i.e., the *p*-value of the SW test of RQRs should be greater than 5%. Whereas under the incorrectly specified model, the null hypothesis should be rejected, so RQR should not be normally distributed, i.e., the *p*-value of the SW test should be less than 5%.

## Simulation studies

The simulation study is carried out to investigate the behavior of the hurdle versus ZI models. The simulation settings consist of model comparison using AIC and Vuong test as well as the overall model goodness of fit calculated as the SW normality test *p*-value for testing the normality of the RQR as described in Section 3.

### 4.1 Simulation settings

To compare the performance of hurdle and ZI models, we consider simulating data from (1) a HNB as the true model and (2) a ZINB as the true model. To highlight the model performance depending on the type of covariates included in the model, we incorporate different types of covariate in the model, i.e., (i) a binary covariate *x* simulated from a Bernoulli distribution *x*∼Bern(*p*) or (ii) a continuous covariate *x* simulated from a Normal distribution *x*∼*N*(0,1). For each simulation scenario, we generated 200 random samples from the true model, and then both HNB and ZINB models are fitted to the simulated datasets with the covariate entering both the logistic and log-linear components of the models.

### 4.2 Factors considered

We varied the values of the following factors to investigate their influence on the performance of the model fits.

- **Strength of the covariate effects**: The strength of the association between the exposure and outcome, measured by *β*_1_ and *α*_1_. The values were set to -2, -1.5, -0.5, -0.1, 0.1, 0.5, 1.5 and 2 in the simulation.
- **Sample size**: To study the finite sample properties of the models, we considered sample sizes *n*=300,500, and 700.
- **Proportion of excess zeros**: The intercept for the logistic component, *β*_0_ was set as 1 to control the percentage of zeros and ensure the simulated datasets are zero-inflated. The intercept of the log-linear component was set as one so that the mean of the counts is not too low. The overdispersion parameter for the NB model is set as 1.2. To confirm the simulated datasets are zero-inflated, we compared the ZINB (true) and NB models in terms of AICs and Vuong’s test for each simulated dataset. The results showed that the true model outperformed the NB model in all simulated datasets.

### 4.3 Evaluation criteria

The performance of the two models is assessed by the relative fit measures and absolute fit measures as follows.

#### 4.3.1 Relative fit measures

For relative fit measures, we used AIC to compare the true and misspecified models in terms of the percentage of the differences in the AICs for the misspecified model and true model are greater than 4 (*%ΔAIC*>4) (Burnham and Anderson 2004) and the mean of the differences of AICs between the misspecified and true model ($\bar {\Delta }$AIC), where *Δ*AIC=AIC(W)-AIC(T), where W and T represent the wrong and true models, respectively. Both measurements should increase as the difference between the true and wrong models increases. We have also used Vuong’s test to compare the ZI and HNB models by measuring the percentage of Vuong test *p*-value <5*%* over repeated samples.

#### 4.3.2 Absolute fit measures

For absolute fit measures, we used the SW normality test to test the normality of RQR in terms of the type I error rates and power. The type I error rates are estimated using the proportion of datasets for which the null hypothesis (true model) is falsely rejected, i.e., the percentage of SW test *p*-value <5*%* for the true model over repeated samples. The power of the test is calculated as the proportion of datasets for which the alternative hypothesis (wrong model) is rejected, i.e., the percentage of SW test *p*-value <5*%* for the wrong model over repeated samples.

### 4.4 Results

#### 4.4.1 Simulation setting *#*1 (True model: HNB)

**Fig. 5:**
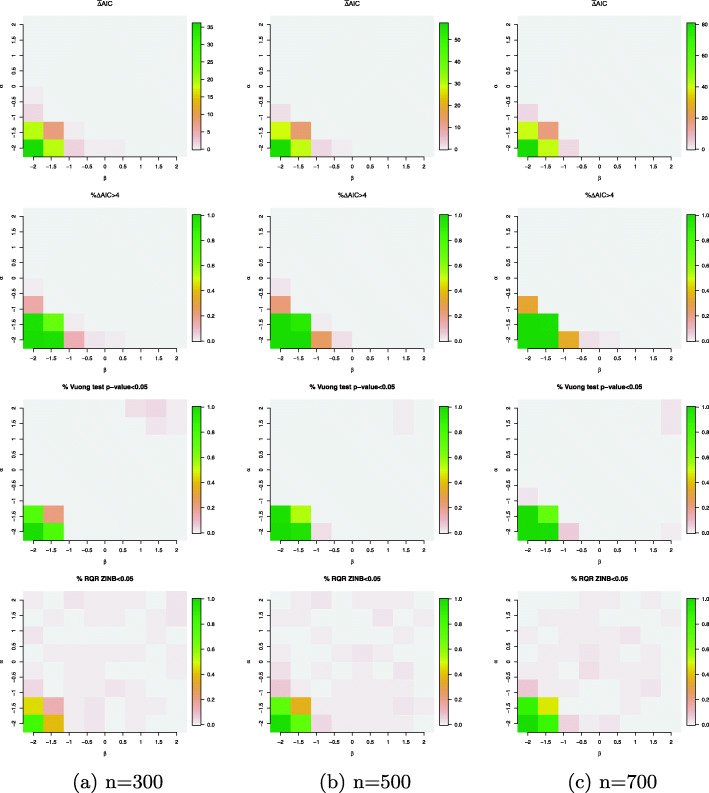
Simulation results for the simulation setting *#*1 (true model: HNB model with a single binary covariate generated from a Bernoulli distribution with probability parameter 0.5). Evaluation criteria include $\bar {\Delta }$AIC (mean difference in AICs of the ZINB and HNB models); *%ΔAIC*>4 (percentage of the differences in AICs between the ZINB and HNB models that are above 4; percentage of Vuong’s test *p*-value <5*%* and percentage of the SW normality test of the RQRs for the ZINB model <5*%*

First, we evaluate the performance of ZINB and HNB models when the data are simulated from a HNB model. Figure 5 plots the relative fit measures and absolute fit measures when the data are simulated from a HNB with a single binary covariate generated from a Bernoulli distribution with probability parameter 0.5. In both the logistic and log-linear components, the regression coefficients vary between -2 to 2 at sample size *n*=300,500 and 700. Our simulation results demonstrate that when the data contains zero-deflated data points as depicted in the left panel of Fig. 1, the ZINB model performs poorly as compared with the counterpart HNB model, yielding a higher AIC and significant difference in model fits according to the Vuong’s test (Fig. 5). RQRs are also not normally distributed under the ZINB model as the percentage of zero-deflated data points increases. As expected, the evidence of rejecting the ZINB model becomes stronger as the sample size increases. On the other hand, when the percentage of zero deflation in the data approaches zero, hurdle and ZINB models yield equivalent fits.

**Fig. 6:**
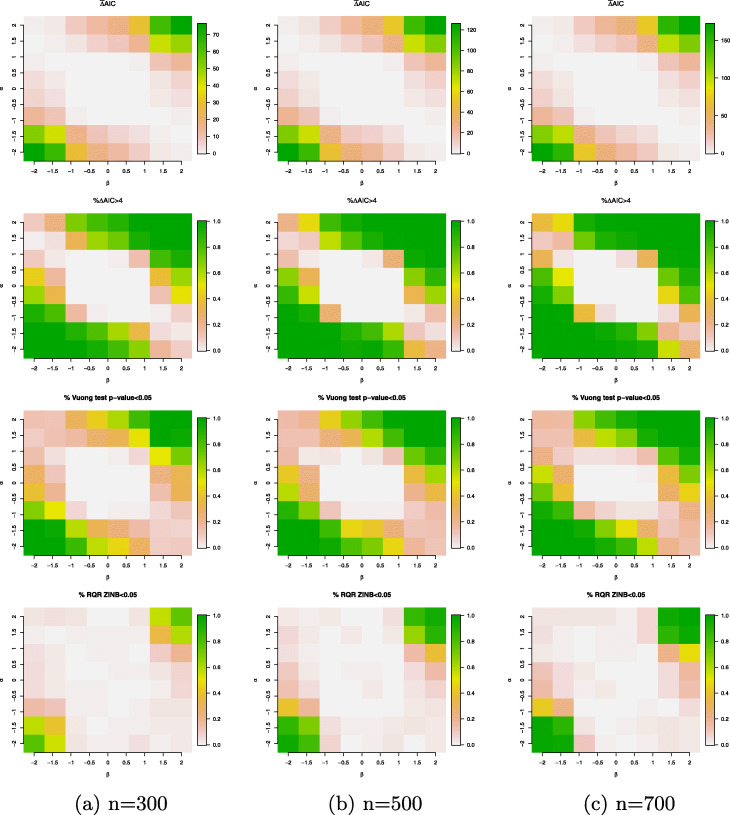
Simulation results for the simulation setting *#*1 (true model: HNB model with a single continuous covariate generated from a standard normal distribution). Evaluation criteria include $\bar {\Delta }$AIC (mean difference in AICs of the ZINB and HNB models); *%ΔAIC*>4 (percentage of the differences in AICs between the ZINB and HNB models that are above 4; the percentage of Vuong’s test *p*-value <5*%* and the percentage of the SW normality test of the RQRs for the ZINB model <5*%*

For the scenario when the data are simulated from a HNB model with a continuous covariate generated from a standard normal distribution, Fig. 6 again confirms that the comparison of the model fits between the HNB and the ZINB model closely align with the percentage of zero-deflated data across all the data points as depicted in the right panel of Fig. 1. More specifically, when there is no zero-deflated data, both HNB and ZINB fit the data equivalently well; whereas, as the percentage of zero deflation increases, the difference in the model fits between the HNB and ZINB models increases. We also remark that the all model comparison measures between the HNB and ZINB models increase as the sample size increases, which suggests that the relative predictive gain by the HNB model increases with increasing sample size. This is not surprising because as the sample size increases, the statistical power of identifying model misspecification increases.

#### 4.4.2 Simulation setting *#*2 (True model: ZINB)

**Fig. 7:**
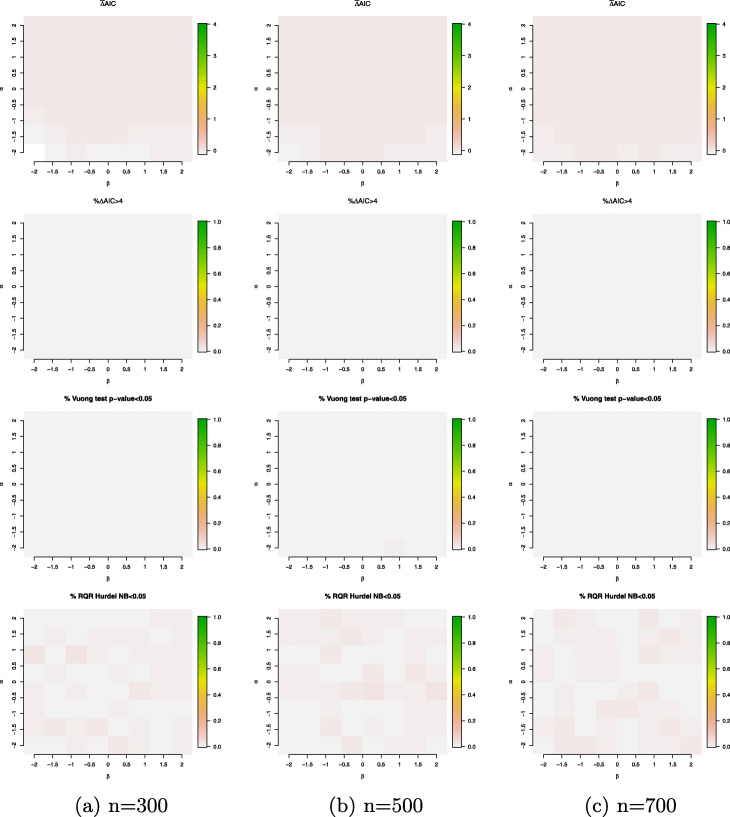
Simulation results for the simulation setting *#*2 (true model: ZINB model with a single binary covariate generated from a Bernoulli distribution with probability parameter 0.5). Evaluation criteria include $\bar {\Delta }$AIC (mean difference in AICs of the HNB and ZINB models); *%ΔAIC*>4 (percentage of the differences in AICs between the HNB and ZINB models that are above 4; the percentage of Vuong’s test *p*-value <5*%* and the percentage of the SW normality test of the RQRs for the HNB model <5*%*

In the second simulation setting, we compare the overall goodness of fit between the ZINB and HNB model when the data are simulated from a ZINB model. Figure 7 plots the relative and absolute fit measures when the data are simulated from a ZINB model containing a single binary covariate generated from a Bernoulli distribution with probability parameter 0.5. In both the logistic and log-linear components, the regression coefficients of the covariate vary between -2 to 2 at sample size *n*=300,500 and 700. Our simulation results demonstrate HNB model can govern the prediction equivalently well as the ZINB model in all scenarios. Recall as shown in the left panel of Fig. 4, even when the structural zeros and sampling zeros are simulated from two largely different processes, the percentage of the large discrepancies between excessive and sampling zeros is close to zero, which provide strong justification to use the discrepancy measure between excessive and sampling zeros developed in Section 2.3.2 to characterize the feature of a ZI model as compared to a hurdle model.

**Fig. 8:**
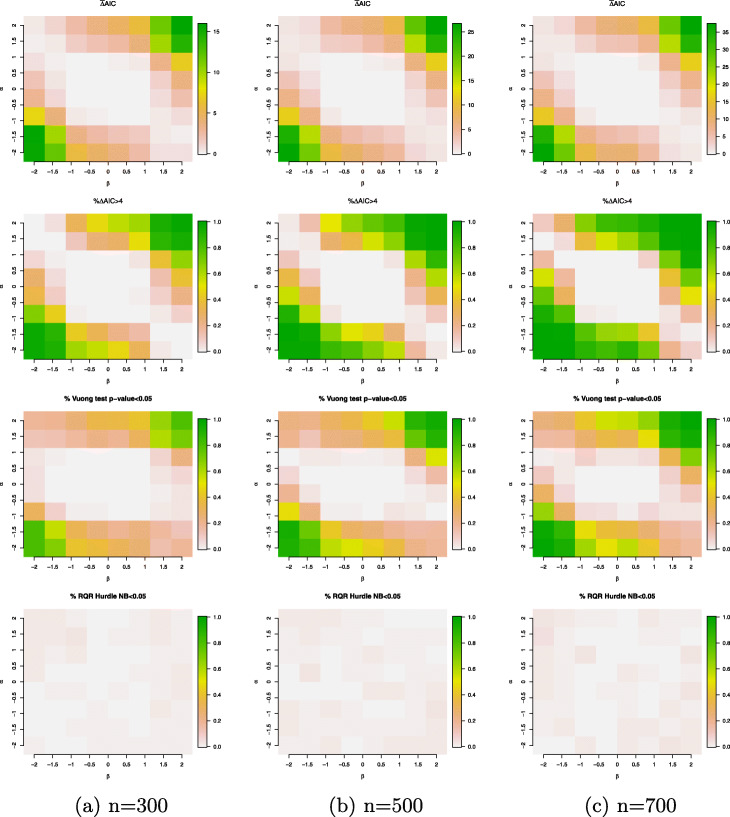
Simulation results for the simulation setting *#*2 (true model: ZINB model with a single continuous covariate generated from a standard normal distribution). Evaluation criteria include $\bar {\Delta }$AIC (mean difference in AICs of the HNB and ZINB models); *%ΔAIC*>4 (percentage of the differences in AICs between the HNB and ZINB models that are above 4; the percentage of Vuong’s test *p*-value <5*%* and the percentage of the SW normality test of the RQRs for the HNB model <5*%*

In the setting when the data are simulated from a ZINB model with a continuous covariate generated from a standard normal distribution, the differences between HNB and ZINB model are observed in Fig. 8. The simulation scenarios where the differences occur are consistent with the settings identified for the large differences between the excessive zeros and sampling zeros as shown in the right panel of Fig. 4. More specifically, the ZINB model has a better fit to the data than the HNB model according to the relative fit measures; whereas, RQRs did not significantly identify inadequacy of the HNB model. This indicates that RQRs are not sensitive to detect small differences between the two models.

## Conclusion and future work

This study reviewed ZI and hurdle models, which are commonly used for modeling zero-inflated count data. This study provided a better understanding of the differences between these two types of models regarding their characteristics and overall model fits.

Our simulation study showed that, with zero-inflated data, zero deflation could occur at certain levels of the covariate, in which case, the hurdle model tends to outperform the ZI model, since only the hurdle model can handle zero-deflated data. Such evidence becomes stronger as the proportion of data points that are zero-deflated increases. Therefore, if there exist a group of subjects in the data with fewer zeros than the sampling zeros from a conventional counts regression model, the hurdle model may be more appropriate than a ZI model. Hurdle and ZI models perform almost equivalently in the overall model fit when there are no or few zero deflations across all the data points when the data are simulated from a hurdle model.

Alternatively, when the data are simulated from a ZI model, the ZI model is more favorable than the hurdle model when there are substantial differences between the probability of structural zeros and sampling zeros. This phenomenon is more evident when the model contains a continuous covariate from a standard normal distribution as compared to the model containing only a single binary covariate. If the processes of generating sampling zeros and structural zeros are not substantially different, the two models yield almost identical model fits.

Overall, our simulation studies indicate the inappropriate application of the ZI and hurdle models could have an undesirable impact on overall model fit. The performances of the two types of models depend on the percentage of the zero-deflated data points in the data and the discrepancy in the data generating processes between the structural zeros and sampling zeros. It is therefore important to recognize the distinct features of these two types of models.

In the current research, we only considered a single covariate to illustrate the model performance depends on the type of covariates included in the model. Additional research needs to be conducted to expand these results to models with multiple covariates. Further, our simulation study only considered independent data. It would be an interesting research topic to consider various correlation structures in the data to assess if the strength of the correlation and correlation structure play a role in choosing between ZI and hurdle model.

## Data Availability

The R codes for the simulation study are available from the corresponding author.

## Abbreviations

ZI: Zero-inflated
ZIP: Zero-inflated poisson
NB: Negative binomial
ZINB: Zero-inflated negative binomial
HNB: Hurdle negative binomial
PMF: Probability mass function
CDF: Cumulative distributions function
RQR: Randomized quantile residuals
SW: Shaprio-Wilk
AIC: Akaike information criterion
